# Predicting prognosis and immune responses in hepatocellular carcinoma based on N7-methylguanosine-related long noncoding RNAs

**DOI:** 10.3389/fgene.2022.930446

**Published:** 2022-08-30

**Authors:** Yu-yang Dai, Yi-ping Gao, Lin-xin Chen, Jin-song Liu, Cheng Zeng, Jian-dong Zhou, Hong-lin Wu

**Affiliations:** ^1^ Department of Radiology, Wujin Hospital Affiliated with Jiangsu University, Changzhou, Jiangsu Province, China; ^2^ Department of Radiology, The Wujin Clinical College of Xuzhou Medical University, Changzhou, Jiangsu, China; ^3^ Department of Interventional Radiology, Nanfang Hospital Affiliated to Southern Medical University, Guangzhou, Guangdong, China; ^4^ State Key Laboratory of Ophthalmology, Optometry and Vision Science, Eye Hospital, School of Ophthalmology and Optometry, School of Biomedical Engineering, Wenzhou Medical University, Wenzhou, Zhejiang, China; ^5^ Department of Oncology, Wujin Hospital Affiliated with Jiangsu University, Changzhou, Jiangsu, China; ^6^ Department of Oncology, The Wujin Clinical College of Xuzhou Medical University, Changzhou, Jiangsu, China; ^7^ Department of Nephrology, Wujin Hospital Affiliated with Jiangsu University, Changzhou, Jiangsu, China; ^8^ Department of Nephrology, The Wujin Clinical College of Xuzhou Medical University, Changzhou, Jiangsu, China

**Keywords:** immune responses, hepatocellular carcinoma, N7-methylguanosine, lncRNA, prognosis

## Abstract

**Background:** Hepatocellular carcinoma (HCC), which has high rates of recurrence and metastasis and is the main reason and the most common tumor for cancer mortality worldwide, has an unfavorable prognosis. N7-methylguanosine (m7G) modification can affect the formation and development of tumors by affecting gene expression and other biological processes. In addition, many previous studies have confirmed the unique function of long noncoding RNAs (lncRNAs) in tumor progression; however, studies exploring the functions of m7G-related lncRNAs in HCC patients has been limited.

**Methods:** Relevant RNA expression information was acquired from The Cancer Genome Atlas (TCGA, https://portal.gdc.cancer.gov), and m7G-related lncRNAs were identified via gene coexpression analysis. Afterward, univariate Cox regression, least absolute shrinkage and selection operator (LASSO) regression, and multivariate regression analyses were implemented to construct an ideal risk model whose validity was verified using Kaplan–Meier survival, principal component, receiver operating characteristic (ROC) curve, and nomogram analyses. In addition, the potential functions of lncRNAs in the novel signature were explored through Gene Ontology (GO) and Kyoto Encyclopedia of Genes and Genomes **(**KEGG) analyses and gene set enrichment analysis (GSEA). At last, in both risk groups and subtypes classified based on the expression of the risk-related lncRNAs, we analyzed the immune characteristics and drug sensitivity of patients.

**Results:** After rigorous screening processes, we built a model based on 11 m7G-related lncRNAs for predicting patient overall survival (OS). The results suggested that the survival status of patients with high-risk scores was lower than that of patients with low-risk scores, and a high-risk score was related to malignant clinical features. Cox regression analysis showed that the m7G risk score was an independent prognostic parameter. Moreover, immune cell infiltration and immunotherapy sensitivity differed between the risk groups.

**Conclusion:** The m7G risk score model constructed based on 11 m7G-related lncRNAs can effectively assess the OS of HCC patients and may offer support for making individualized treatment and immunotherapy decisions for HCC patients.

## 1 Introduction

Primary hepatic carcinoma, the sixth most common malignant neoplasm and the third most common cause of cancer mortality worldwide, includes hepatocellular carcinoma (HCC) (which accounts for 75%–85% of cases) and other kinds of hepatic cancer (Sung et al., 2021).

HCC is notoriously characterized by poor prognosis with locoregional treatments such as resection, percutaneous ablation, transarterial chemoembolization (TACE), and radioembolization (European Association for the Study of the Liver, 2018). Owing to the steady and broad resistance of HCC to cytotoxic chemotherapy, immunotherapy has recently been recommended as an option for HCC cases expressing various tumor-associated antigens. However, no studies have shown efficacy (Prieto et al., 2015). Systemic therapy has been the default therapy for years, but the current first-line drug, sorafenib, a multi-tyrosine kinase inhibitor, does not substantially prolong the overall survival (OS) of patients with advanced- or moderate-stage HCC (Llovet et al., 2015).

The immune system has a considerable influence on tumor progression. The liver contains a variety of stromal cells and various immunoinhibitory substances that enable it to function as a tolerogenic immune organ to avoid adverse reactions to chronic pathogen exposure (Pardee and Butterfield, 2012). Immune checkpoints and their ligands are expressed on the surface of various effector lymphocytes (Heinrich et al., 2018; Inarrairaegui et al., 2018).

In recent years, gene signatures have been developed and found to have predictive value (Jiao and Wang, 2016). Almost 60,000 human genes can be transcribed by the genome, with approximately 20,000 being protein-coding genes and the rest being noncoding genes, including approximately 16,000 long noncoding RNAs (lncRNAs) (Rinn and Chang, 2012). Research attention has shifted toward lncRNAs because they have shown promising characteristics, such as being important in regulating biological events involving cell proliferation and apoptosis (Gonzalez et al., 2015; Ransohoff et al., 2018; Simion et al., 2019). LncRNAs recognize proteins, operate as molecular sponges to disrupt microRNA interactions, change the epigenome, and affect gene expression by binding to gene promoters (Fu, 2014; Huang, 2018). A series of studies have probed the prognostic capacity of signatures based on lncRNAs in HCC, and the results of such studies will be beneficial for clarifying relevant molecular mechanisms. For example, nine lncRNAs related to ferroptosis-mediated programmed cell death and clinical information have been proven to predict patient prognosis in HCC (Xu Z. et al., 2021). In addition, a lncRNA signature (APBB1-1, FBXO42-1, JAKMIP2-1, and MMADHC-5) related to the regulation of proliferation and lipid metabolism could effectively predict prognosis in HCC patients (Shi et al., 2021).

Furthermore, RNA modification greatly influences the regulation of gene expression (He, 2010), and many RNA modifications have been linked to vital biological pathways, particularly in cancer cells (Aas et al., 2003; Shimada et al., 2008; Shimada et al., 2009).

However, research on the posttranscriptional modification of lncRNAs is lacking. In eukaryotes, N7-methylguanosine (m7G), a frequent posttranscriptional alteration of mRNA and lncRNA, is needed for efficient gene expression and cell properties (Juhling et al., 2009; Tomikawa, 2018). Although increasing evidence suggests that m7G modification is linked to the onset and course of various illnesses, the RNA m7G methylation profile in HCC has yet to be revealed.

Given the potential of immunotherapy and the toned to rapidly identify novel and reliable screening methods that can enhance the diagnosis of and prediction of therapeutic benefit in HCC, here, a prospective prognostic model based on m7G-related lncRNAs was established. This model might help predict which patients will benefit from immunotherapy.

## 2 Materials and methods

### 2.1 Data acquisition and manipulation

LncRNA expression information from 424 samples, including tumor and normal liver tissues, was retrieved from The Cancer Genome Atlas (TCGA) up to 1 February 2022. The clinical characteristics of patients with an overall survival time exceeding 30 days and sufficient clinical information were also retrieved, and the clinical information of samples is provided in Supplementary Table S1. m7G genes were retrieved from the Molecular Signatures Database (MSigDB, http://www.gsea-msigdb.org) and previous literature (Subramanian et al., 2005; Liberzon et al., 2015), and the gene set can be found in Supplementary Table S2. All the data were transformed into FPKM values to simplify downstream analysis. In addition, because all the data are publicly available, ethics approval and consent were deemed unnecessary.

### 2.2 Identification of N7-methylguanosine–related long noncoding RNAs

Pearson correlation was employed to evaluate the association between m7G-related genes and lncRNAs. Those lncRNAs that met the criteria (|Pearson R| > 0.4 and *p* < 0.001) were identified as m7G-associated lncRNAs (Supplementary Table S3). The samples were then randomly divided into two groups: 70% of the total samples were allocated to the training dataset, whereas 30% were allocated to the testing dataset.

### 2.3 Model construction

Univariate Cox regression analysis was implemented to recognize m7G-related lncRNAs associated with the OS of HCC patients in the training set. The remaining was selected further using the least absolute shrinkage and selection operator (LASSO)-penalized Cox regression analysis, which aims to strengthen the predictive value and applicability of the prognostic model, as well as alternative methods and normalization were used. This approach has been widely used to minimize overfitting and identify the best features in high-dimensional data with low correlation and substantial predicted value. As a result, this strategy can efficiently identify the best predictive factors and provide prognostic indicators for predicting clinical outcomes.

At last, we further utilized multivariate Cox regression analysis to build an m7G-associated lncRNA risk model for predicting OS. The following mathematical equation was utilized to compute the m-7G risk score:

$$\text{risk}\;\text{score}=\;\overset{n}{\underset{i=1}{\sum }}Coef\left(i\right)\text{∗}lncRNAexpr\left(i\right)$$

where n, coef(i), and expr(i) represent the number, homologous coefficient, and FPKM value of a given risk-related lncRNA, respectively.

The results of univariate Cox analysis, LASSO penalized Cox regression analysis, and multivariate Cox analysis are shown in Supplementary Table S4.

### 2.4 Independent dataset validation

Univariate Cox regression and multivariate Cox regression analyses were applied to validate the utility of the selected lncRNAs in the entire dataset, and relative clinical information was obtained from TCGA. A *p*-value of 0.05 was used to identify relevant independent predictive variables.

### 2.5 Nomogram construction and performance analysis

To improve the model’s accuracy, we used the R package “rms” to create a nomogram model based on the stage and risk score with independency and used this model to calculate risk scores.

### 2.6 Kaplan–Meier survival analysis

Kaplan–Meier (K-M) analysis was performed using a log-rank test to investigate the predictive value of the m7G-lncRNA signature in HCC patients separated into distinct groups. Result with a *p*-value < 0.05 were considered significant.

### 2.7 Principal component analysis

Principal component analysis (PCA) was implemented to validate further the accuracy of the m7G-related lncRNA model in grouping samples based on differences in gene expression profiles.

### 2.8 Functional enrichment analysis

Gene Ontology (GO) and Kyoto Encyclopedia of Genes and Genomes (KEGG) enrichment analyses were adopted using the R package “cluster Profiler” to determine potential differentially enriched biological functions and pathways between the two risk groups. Gene set enrichment analysis (GSEA) investigated underlying functions associated with the obtained differentially expressed genes. Genes with normalized *p*-value < 0.05 and FDR < 0.25 were included in the enriched gene set.

### 2.9 Nonnegative matrix factorization

Unsupervised consensus clustering analysis using the R package “Consensus Cluster Plus” was used to identify molecular subgroups based on the risk-related lncRNA expression.

### 2.10 Immunologic function analysis

The XCELL (Aran et al., 2017), TIMER (Li et al., 2017), QUANTISEQ (Hao et al., 2021), MCP-counter (Becht et al., 2016), EPIC (Racle and Gfeller, 2020), CIBERSORT−ABS (Xu Q. et al., 2021), and CIBERSORT (Chen et al., 2018) algorithms were used to assess the levels of various tumor-infiltrating immune cells: endothelial cells, hematopoietic stem cells, common myeloid progenitors, macrophages, activated mast cells, monocytes, central memory CD4^+^ T cells, neutrophils, memory B cells, cancer-associated fibroblasts, plasma B cells, M0 macrophages, M1 macrophages, activated natural killer (NK) cells, nonregulatory CD4^+^ T cells, activated memory CD4^+^ T cells, and so on.

### 2.11 Differentially expressed gene analysis

Differentially expressed genes (DEGs) were identified using the R package “limma” (|log2FC| > 1 and FDR < 0.05).

### 2.12 Drug sensitivity prediction

Using the R package “pRRophetic,” the drug response of HCC patients was predicted. A *p*-value < 0.01 was employed to predict potential treatments for patients in different risk groups.

### 2.13 Statistical analysis

The Chi-square test was conducted to identify differences between groups in various datasets, and the Wilcoxon test was used to compare two groups. In addition, the log-rank test was employed to perform the K-M survival analysis. Results with a *p*-value < 0.05 were considered significant.

## 3 Result

### 3.1 Identification of N7-methylguanosine–related long noncoding RNAs

**FIGURE 1:**
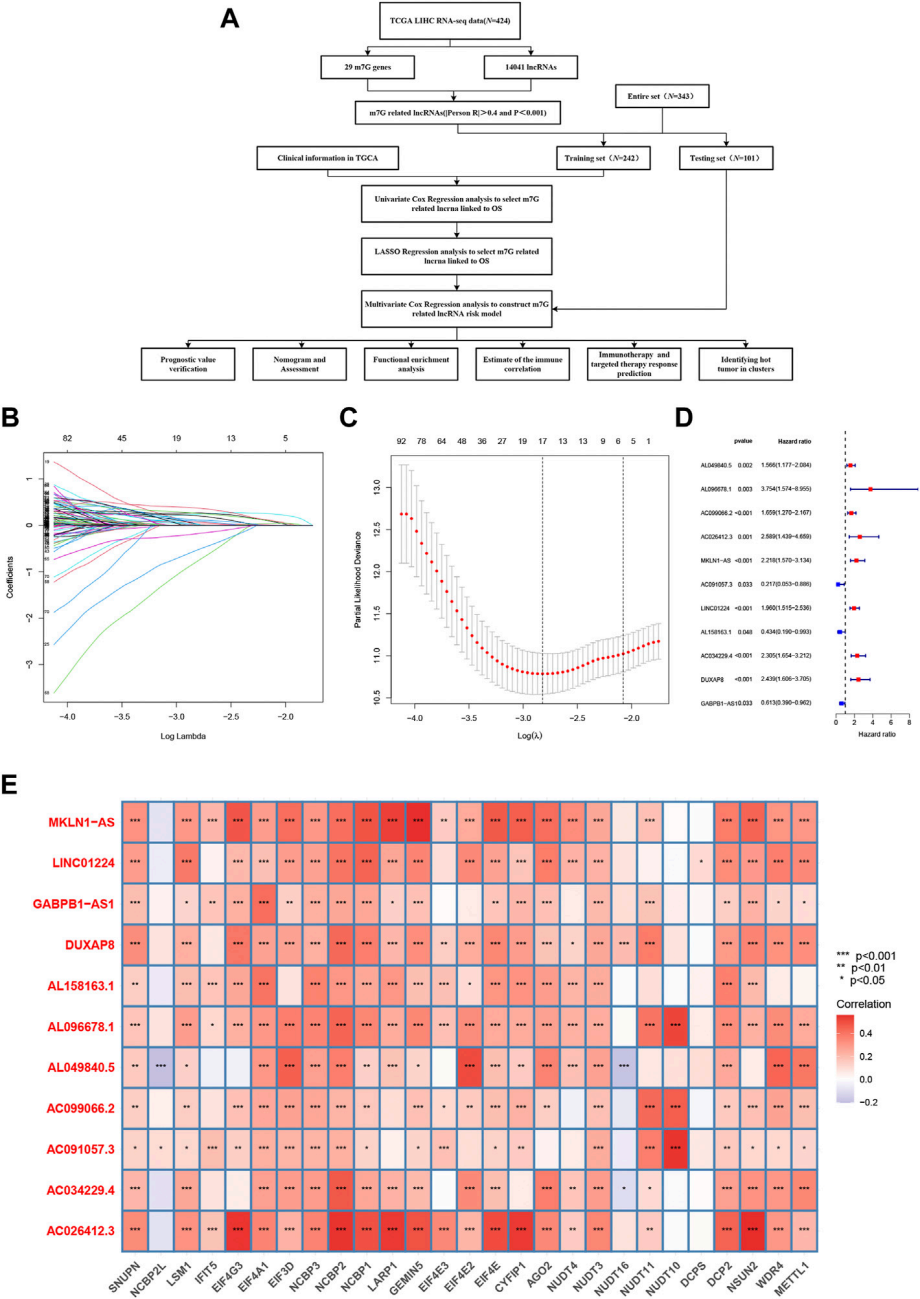
Construction of the model based on N7-methylguanosine (m7G)-related long noncoding RNAs (lncRNAs) in hepatocellular carcinoma (HCC). **(A)** Workflow of the study. **(B)** Prognostic lncRNAs in HCC were selected based on regression coefficient analysis. **(C)** Least absolute shrinkage and selection operator (LASSO) Cox regression with 10-fold cross-validation was used to determine the optimal factors for the HCC cohort. **(D)** Multivariate Cox regression analysis was used to identify prognostic m7G-related lncRNAs to construct an m7G-related lncRNA risk model. **(E)** Correlations between the expression of the differentially expressed lncRNAs and m7G genes in HCC.

A flowchart of the whole research, including the construction of the model and further studies, is depicted in Figure 1A. First, we obtained publicly available HCC patient datasets, including data for 14,041 lncRNAs and 29 m7G genes from TCGA. m7G-associated lncRNAs were characterized as lncRNAs significantly associated with one of the 29 m7G genes.

### 3.2 Construction and validation of a risk model based on N7-methylguanosine–related long noncoding RNAs

#### 3.2.1 Model construction

In total, 276 prognosis-related m7G-related lncRNAs were recognized using univariate Cox regression analysis. These lncRNAs were further evaluated using LASSO penalized Cox regression analysis, resulting in 17 lncRNAs (Figures 1B, C). Multivariate Cox regression analysis was ultimately performed to identify prognosis-related differential lncRNAs, and 11 m7G-related lncRNAs were utilized to construct the m7G-related lncRNA risk model (Figure 1D). Figure 1E shows the correlations of these m7G-related lncRNAs with m7G gene in the TCGA dataset. Hence, a novel prognostic model based on m7G-related lncRNAs was successfully developed to forecast the OS of HCC patients.

Afterward, we attempted to verify this model in the complete dataset and carried out a bioinformatics analysis.

#### 3.2.2 Model validation

**FIGURE 2:**
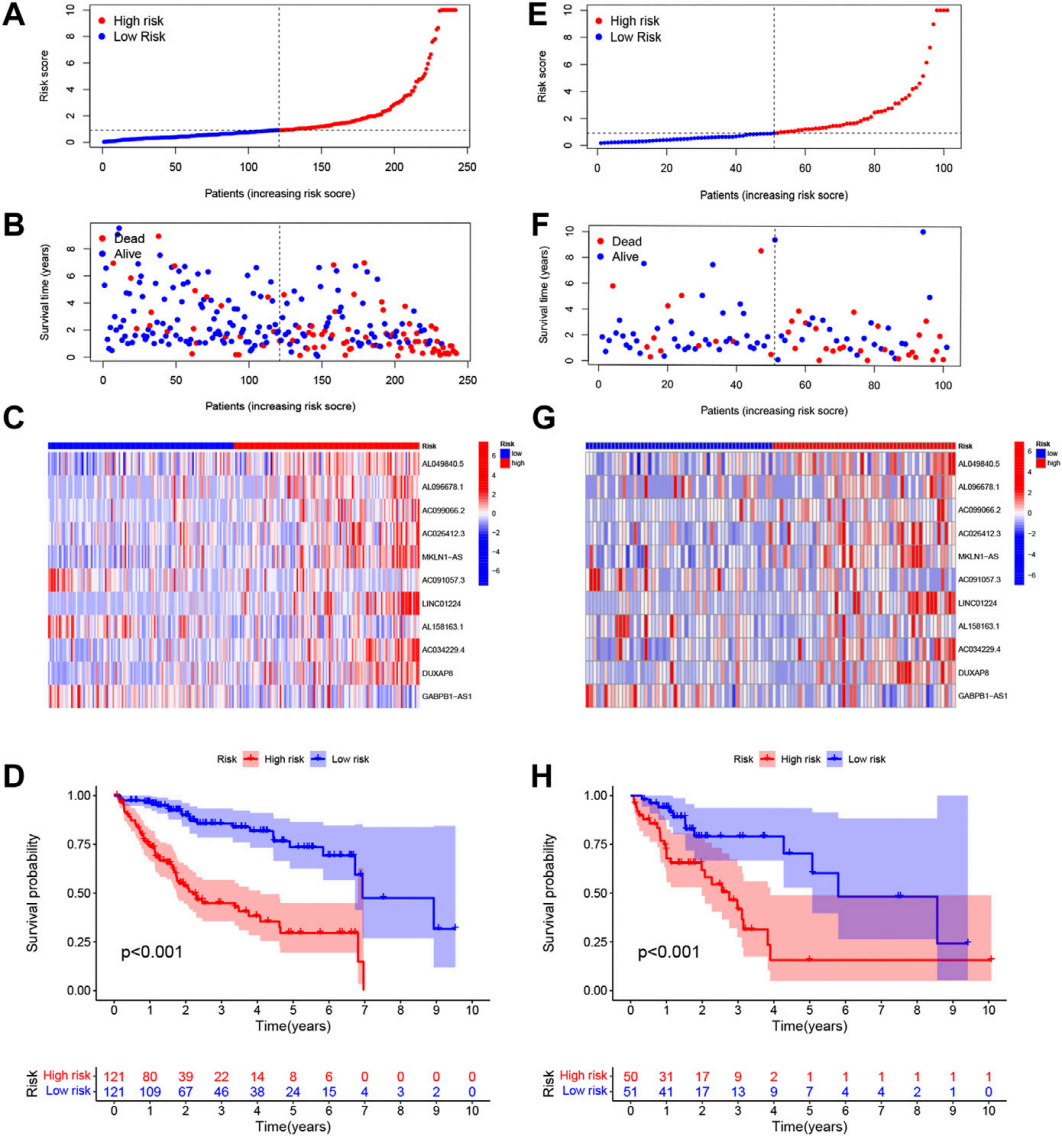
Distributions of the signature risk score, survival status, and expression of relevant long noncoding RNAs (lncRNAs) in the training set **(A–C)** and the validation set **(E–G)**. Kaplan–Meier survival analysis was used for the training set **(D)** and testing set **(H)** analysis.

**FIGURE 3:**
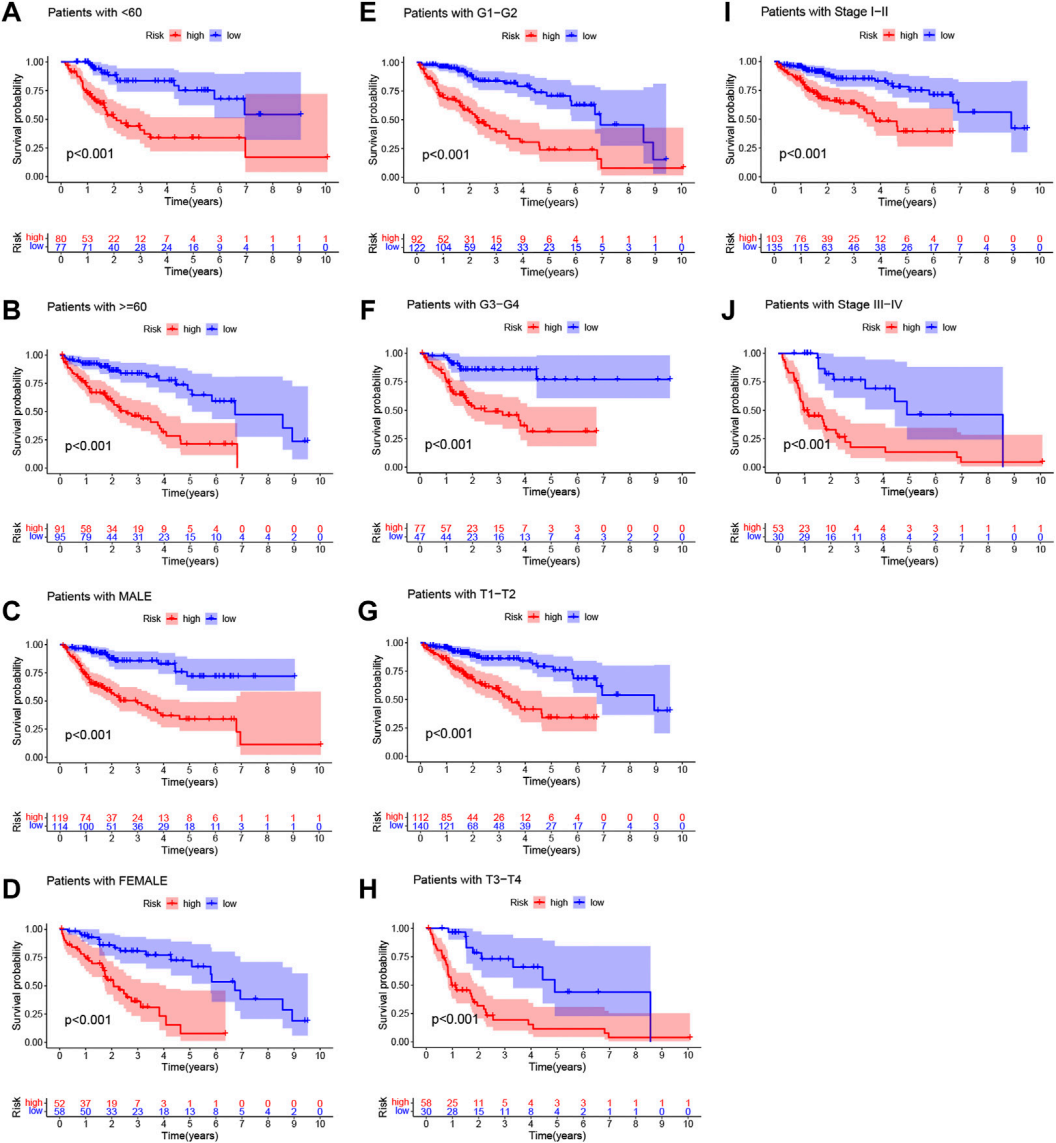
Kaplan‐Meier (K-M) survival analysis to determine whether patient overall survival (OS) was correlated with clinical factors, including age **(A,B)**, sex **(C,D)**, grade**(E,F)**, T stage **(G,H)**, and stage **(I,J)**.

The risk scores of the training set and testing set were calculated, and then the patients were divided into high- and low-risk groups according to the cutoff value of the risk score in the training set, and the median value of the risk score in the training set is identified as the cutoff value. The distributions of the signature risk score, survival status, and expression of relevant lncRNAs in the training set (Figures 2A–C) and the validation set (Figures 2E–G) are depicted in Figure 2. As the risk score increased, the number of deaths among HCC patients increased. Afterward, K-M survival analysis was performed with the training set (Figure 2D) and testing set (Figure 2H), and the results showed that OS was better in the low-risk group in both datasets. In addition, the subgroup K-M survival analysis of patients grouped by different clinical characteristics showed consistent results (Figure 3).

**FIGURE 4:**
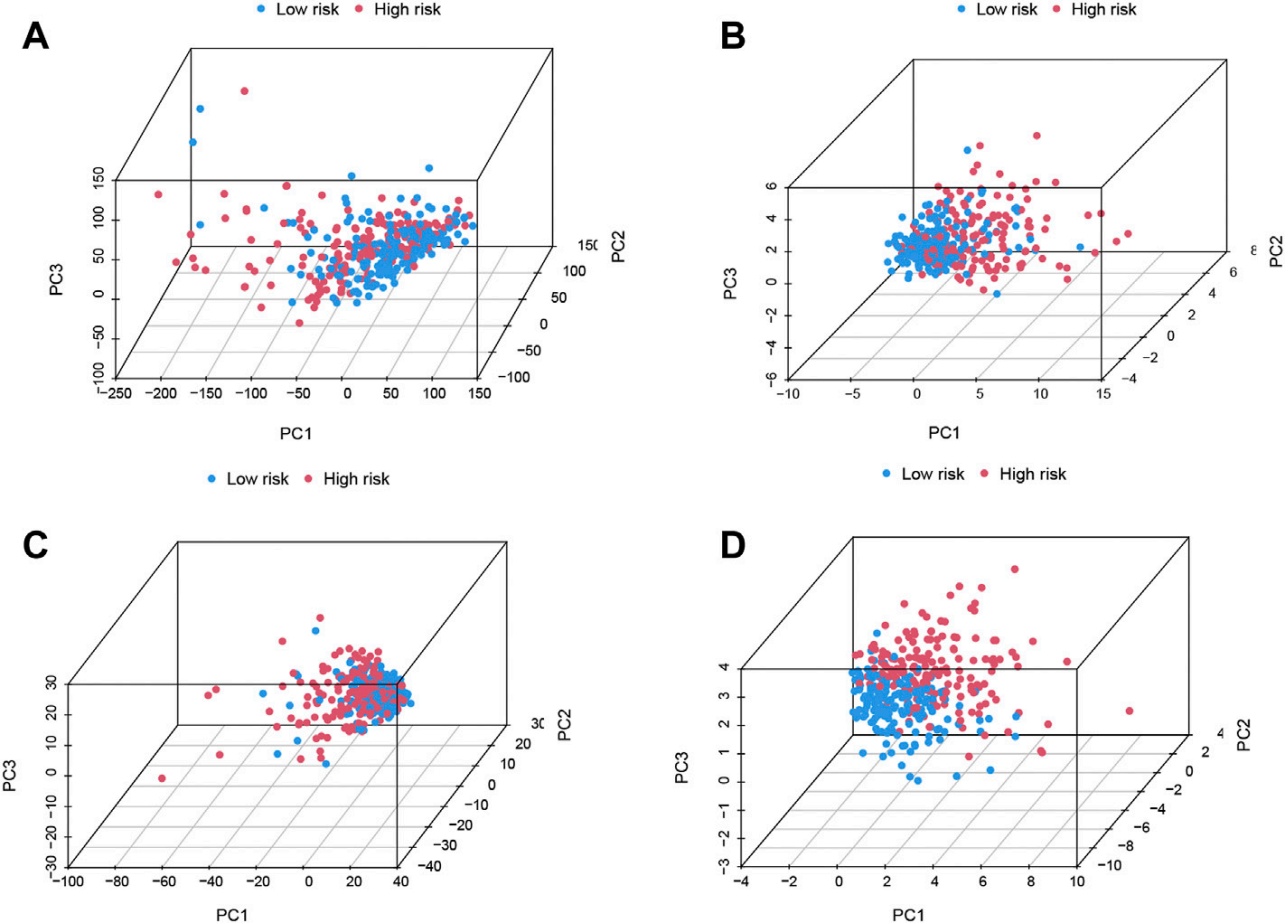
Principal component analysis (PCA) confirms the discriminatory ability of **(A)** total gene expression profiles, **(B)** 29 N7-methylguanosine (m7G) genes, **(C)** m7G-related long noncoding RNAs (lncRNAs), and **(D)** 11 m7G-related risk lncRNAs.

The differences between the two risk groups in total gene expression profiles, the expression of the 29 m7G genes, and the expression of the m7G-related lncRNAs and 11 m7G-related risk lncRNAs in our study were each confirmed using PCA (Figures 4A–D). Figures 4A–C shows random clustering of the risk groups. In comparison, the findings from our model revealed that the two groups had distinct distributions (Figure 4D). These findings imply that the prognostic signature can differentiate patients into two risk grades.

**FIGURE 5:**
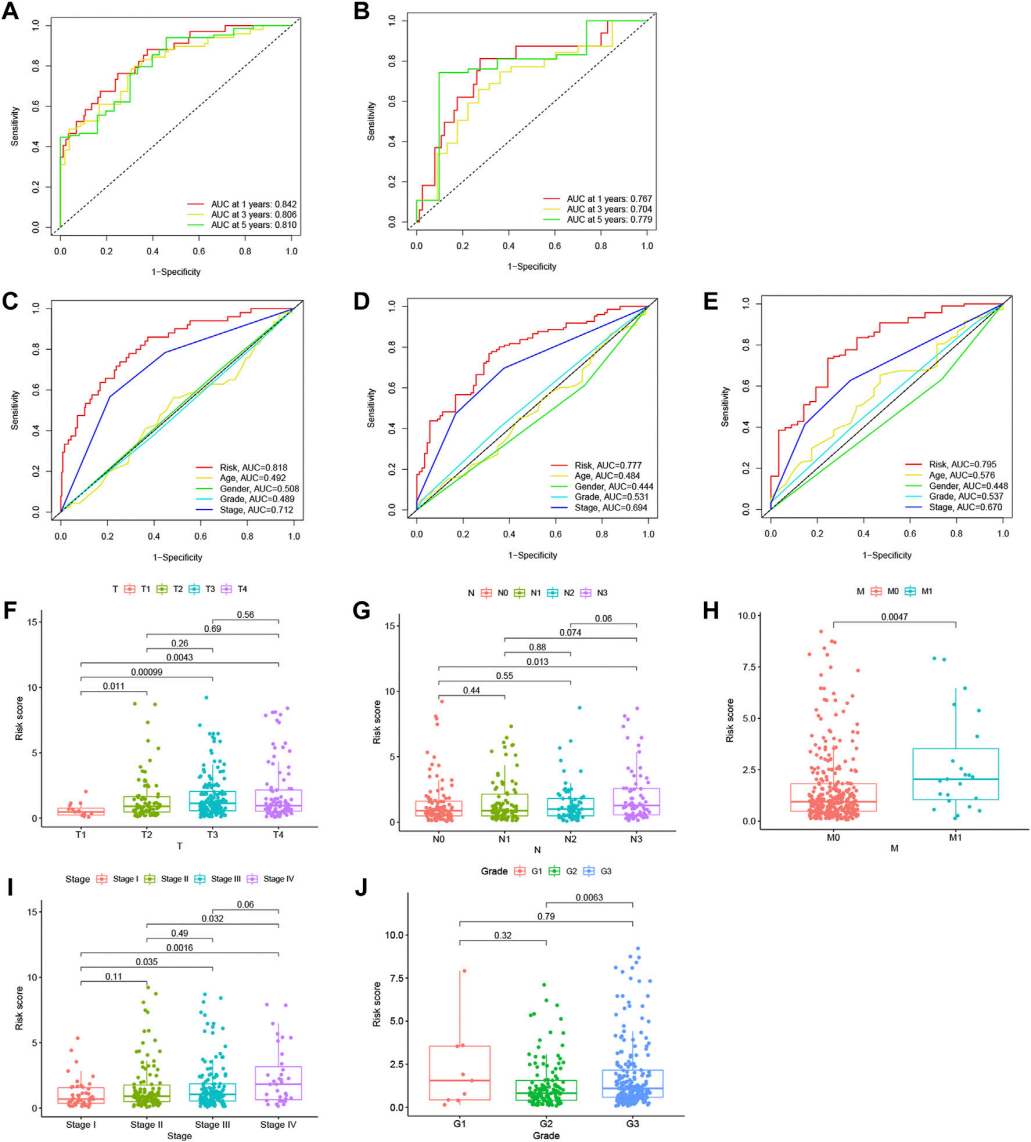
Analysis of the N7-methylguanosine (m7G) risk model and other clinical characteristics. Receiver operating characteristic (ROC) curve analysis was performed, and the acreage under the curve (AUC) for 1-, 3-, and 5-year overall survival (OS) was calculated in the training dataset **(A)** and in the testing dataset **(B)** to verify the accuracy of the long noncoding RNAs (lncRNA) signature. The AUC values based on the entire set of the risk score combined with other clinicopathological factors for predicting 1- **(C)**, 3- **(D)**, and 5-year **(E)** OS demonstrated the reliability of the risk model. Risk score distribution was based on patients’ tumor/node/metastasis (TNM) stage **(F–H)**, stage **(I)**, and grade **(J)**.

The accuracy of this model was assessed in both datasets using receiver operating characteristic (ROC) curve analysis. The acreage under the curve (AUC) values of the training set and validation set for 1-, 3-, and 5-year OS were 0.742, 0.806, and 0.810, respectively (Figure 5A), and 0.767, 0.704, and 0.779, respectively (Figure 5B), indicating that the lncRNA signature could precisely predict the prognosis of HCC patients. Based on the entire set, the AUC of the risk score was comparable to that of other clinical parameters for predicting 1- (C), 3- (D), and 5-year (E) OS, indicating that the 11-m7G-related-lncRNA-based model for predicting HCC prognosis was comparably reliable. The risk score distributions based on patient tumor/node/metastasis stage, stage, and grade are displayed in Figures 5F–J, and the results reveal that this model is helpful in patients with moderate- or advanced-stage disease.

**FIGURE 6:**
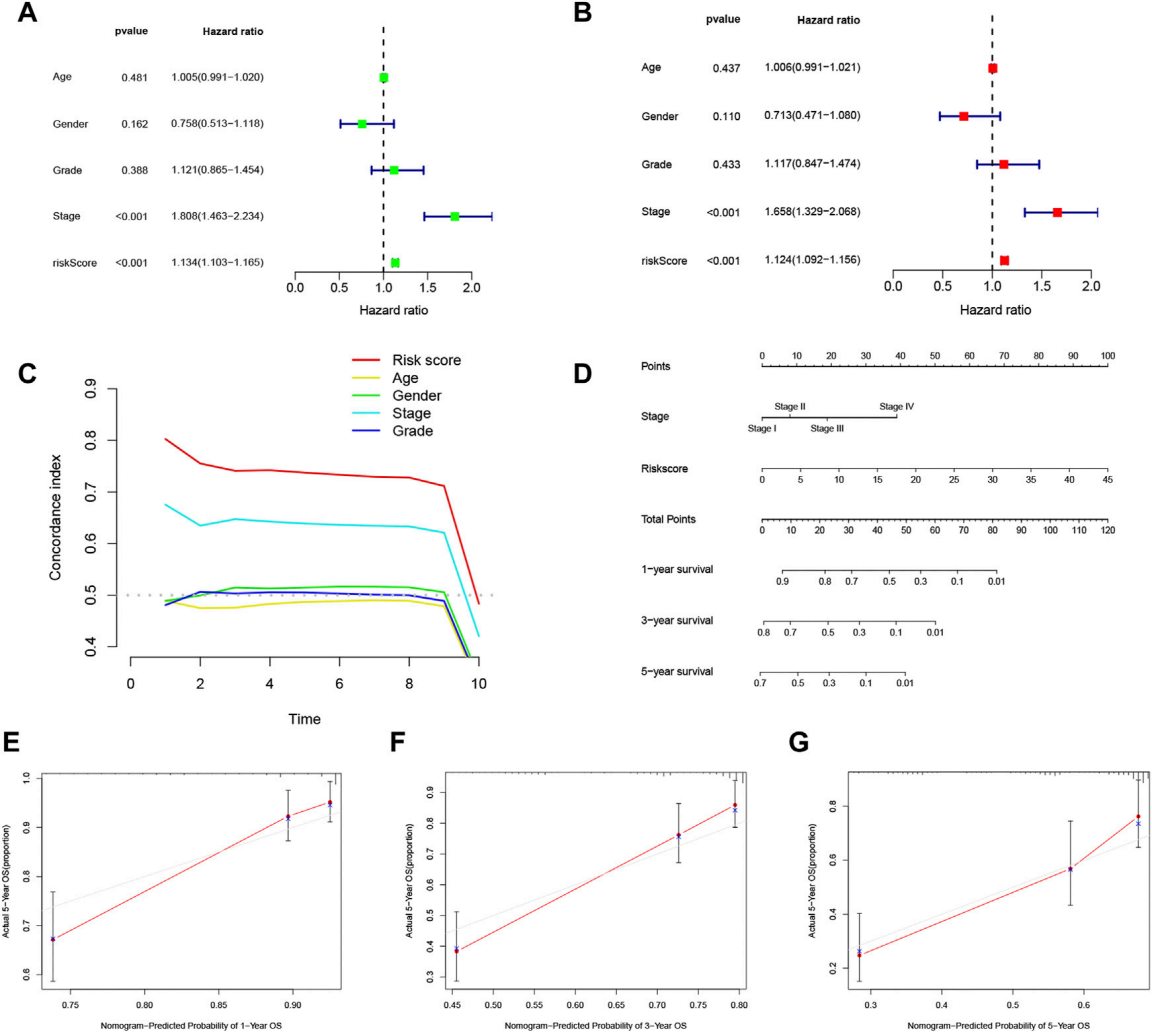
Analysis of the independent predictive ability of the risk score and other factors. **(A,B)** Univariate and multivariate Cox regression analyses were used to determine whether the risk score was an independent factor predicting survival in hepatocellular carcinoma (HCC). **(C)** Concordance index (CI) values were calculated to assess the independent predictive utility of the factors. (**D)** A nomogram including the independent prognostic factors stage and risk score was generated to predict the 1-, 3-, and 5-year overall survival (OS). The 1- **(E)**, 2- **(F)**, and 3-year **(G)** calibration plots are shown.

Univariate Cox regression analysis and multivariate Cox regression analysis were utilized to assess whether the score calculated based on the 11 m7G related lncRNA-based risk model could function as an independent prognostic indicator for HCC patients. In the univariate Cox regression analysis, the risk score (hazard ratio: 1.134, 95% confidence interval: 1.103–1.165, *p* < 0.001) and T stage (hazard ratio: 1.808, 95% confidence interval: 1.463–2.234, *p* < 0.001) were independent predictors of HCC prognosis (Figure 6A). These results demonstrated that not only the lncRNA-based risk score (hazard ratio: 1.124, 95% confidence interval: 1.092–1.156, *p* < 0.001) but also T stage (hazard ratio: 1.658, 95% confidence interval: 1.329–2.068, *p* < 0.001) could independently predict patient OS in HCC (Figure 6B), which indicated that the other clinical variables did not affect the model.

The concordance index (CI) evaluates the predictive capability of independent factors based on the probability of the predicted outcome agreeing with the actual outcome. The CI of the risk score seemed to be higher than those of other clinical parameters, implying that the risk score may better predict the outcome of HCC patients (Figure 6C). To investigate the independence of the risk score as a risk factor, a nomogram based on the independent prognostic parameters stage and m7G risk score was constructed to forecast patient 1-, 3-, and 5-year OS (Figure 6D). The 1-, 3-, and 5-year calibration plots showed good concordance of the nomogram-predicted and actual patient OS values, suggesting that the nomogram can aid planning of short-term follow-up visits for individual treatments (Figures 6E–G). Moreover, the risk score demonstrated the most robust predictive competence among all the clinical variables, consistent with the multivariate Cox regression analysis. The novel m7G-associated lncRNA score was a reliable and independent factor for predicting HCC patient prognosis.

#### 3.2.3 Prediction of immunotherapy benefit

To determine whether the m7G-related prognostic lncRNAs are related to the immune response, Spearman’s correlation analysis was employed to determine the correlation between the risk score and diverse immune functions and infiltrating immune cell levels in the two groups.

**FIGURE 7:**
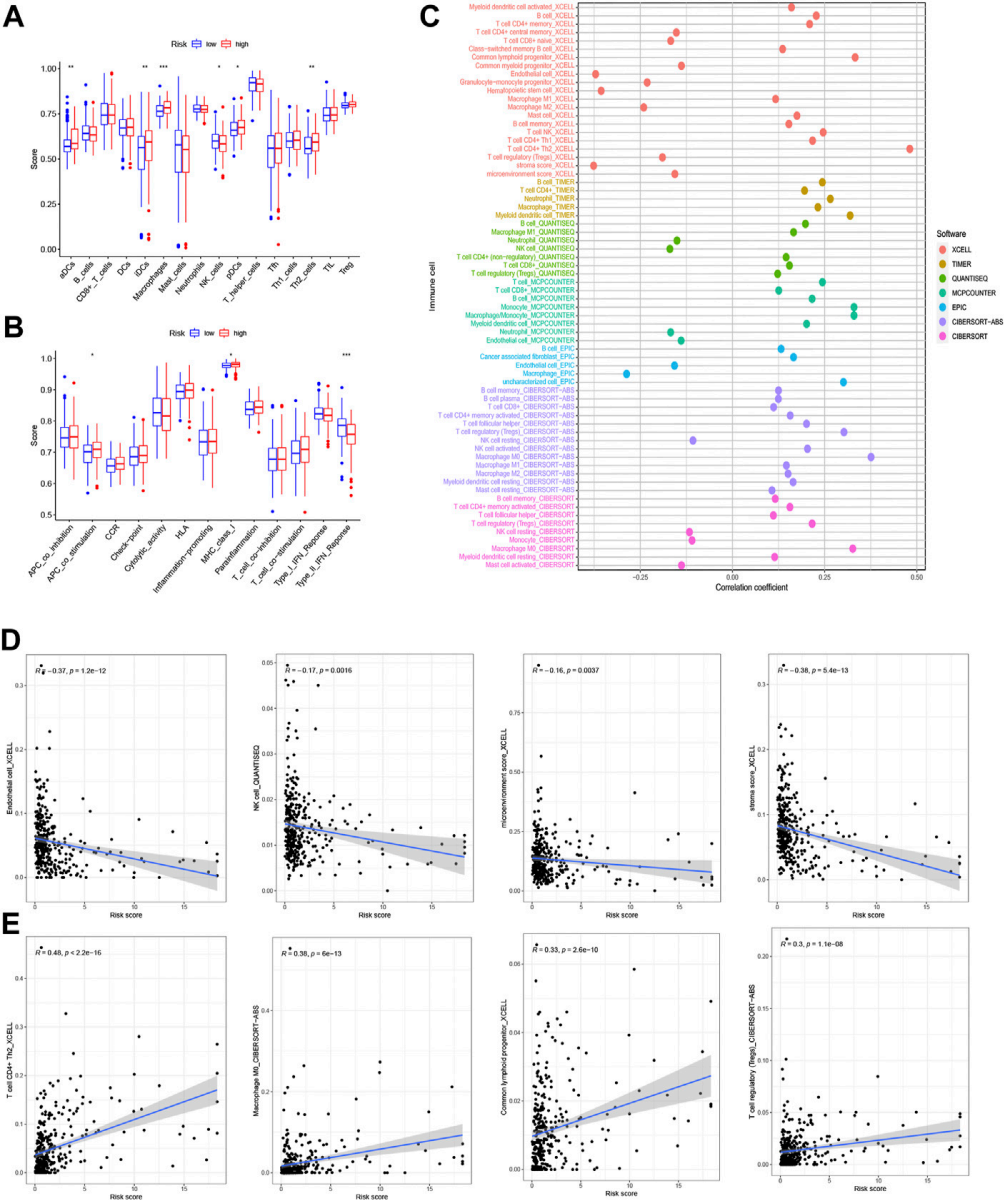
Analysis of immune function. **(A)** Abundances of 22 types of infiltrating immune cells. **(B)** Relationship between immune scores and a series of immune functions. **(C)** The relationship between the risk score and immune cell infiltration in hepatocellular carcinoma (HCC) was assessed using the XCELL, TIMER, QUANTISEQ, MCPCOUNTER, EPIC, CIBERSORT−ABS, and CIBERSORT algorithms. **(D)** Infiltrating immune cells negatively correlated with risk score (endothelial cell, natural killer (NK) cell, microenvironment score, stroma score). **(E)** Infiltrating immune cells positively correlated with risk score (T cells CD4^+^ Th2, macrophages M0, common lymphoid progenitor, T cell regulatory).

We investigated the levels of 22 infiltrating immune cells (Figure 7A). The abundances of antigen-presenting cells (APCs), immature dendritic cells (IDCs), macrophages, plasmacytoid dendritic cells (PDCs), and Th2 cells were lower in the low-risk group, while the NK cell abundance was high.

The relationships between the risk score and various immune functions are depicted in Figure 7B. The score targeting type II interferons (IFN-II) in the low-risk group was significantly higher than that in the high-risk group, while the score with APC costimulation and MHC class I showed an inverse correlation with the risk score. The demonstration for various immune cell infiltration associated with the risk score is in Figure 7D.

The association of the risk score with immune cell infiltration was then evaluated using the XCELL, TIMER, QUANTISEQ, MCPCOUNTER, EPIC, CIBERSORT−ABS, and CIBERSORT algorithms (Figure 7C and Supplementary Table S5). The immune cell infiltration score was shown to be inversely correlated with the abundances of endothelial cells, NK cells, the microenvironment score, and the stroma score (Figure 7D), and the levels of CD4^+^ T cells Th2, M0 macrophages, common lymphoid progenitors, and regulatory T cells (Tregs) showed a surprising positive relationship with the risk score (Figure 7E) (R > 0.1, all *p* < 0.05). Immune cell subtype infiltration may have a significant impact on prognosis.

#### 3.2.4 Gene enrichment and alteration analysis based on the risk score

**FIGURE 8:**
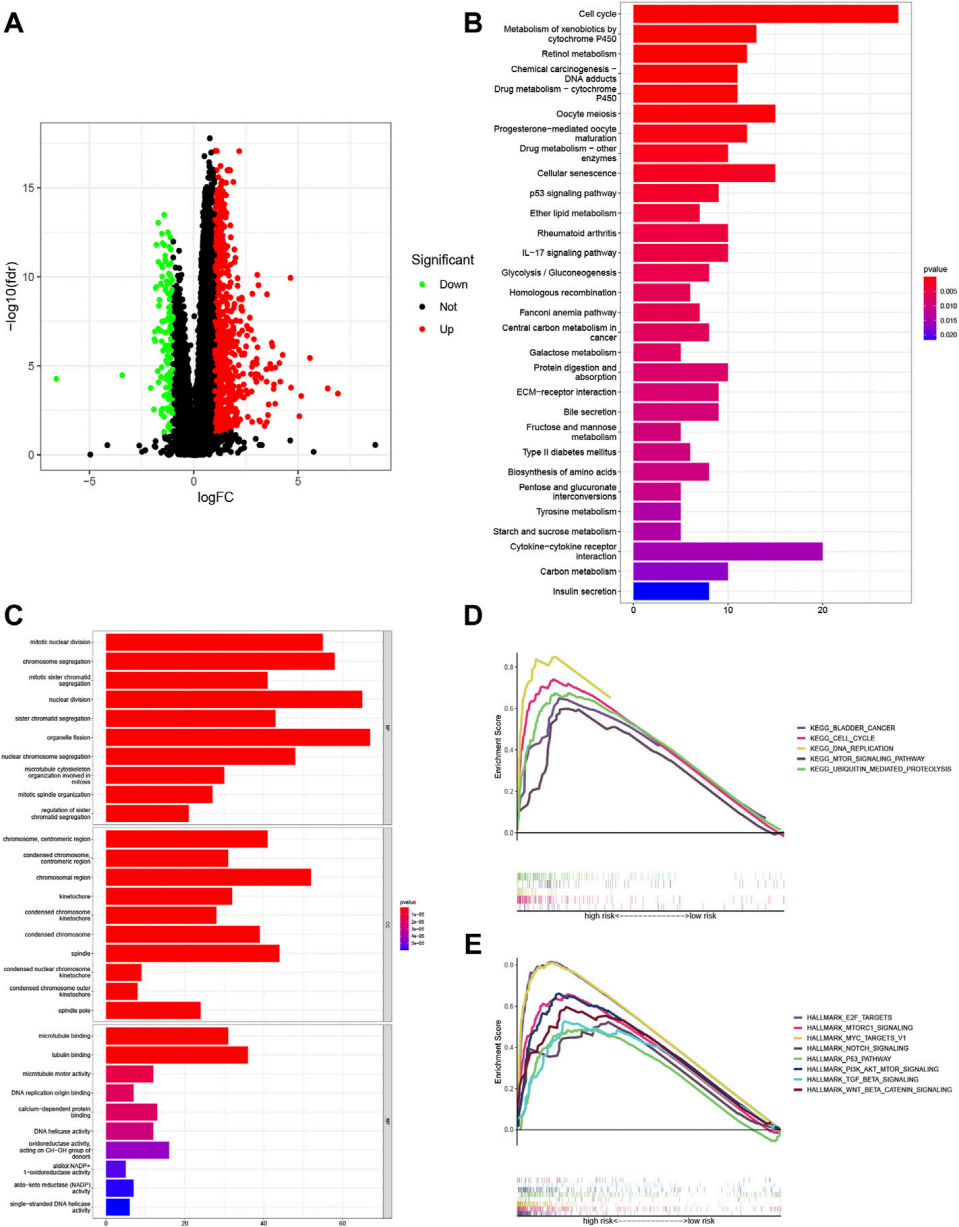
Enrichment analyses of the potential functions of the differentially expressed genes (DEGs). **(A)** Volcano plots showing the relationships of the significant differentially expressed long noncoding RNAs (lncRNAs) with upregulated, downregulated, and nondifferentially expressed genes represented by red, blue, and black, respectively. Underlying pathways and functions of the signature lncRNAs were predicted with Gene Ontology (GO) enrichment analysis **(B)** and Kyoto Encyclopedia of Genes and Genomes (KEGG) pathway analysis **(C)**. Gene set enrichment analysis (GSEA) of the KEGG pathway terms **(D)** and HALLMARK pathway analysis **(E)** further revealed the possible roles of lncRNAs in the signature in the tumor.

A volcano plot was generated to show the significantly altered genes between groups with different risk scores. Significant DEGs were selected based on the criteria of |log2FC| > 1 and FDR < 0.05 (Supplementary Table S6). Significantly upregulated, downregulated, and nondifferentially expressed lncRNAs are represented by red, blue, and black, respectively (Figure 8A).

GO enrichment analysis and KEGG pathway analysis were adopted to investigate the mechanisms linking the risk signature with the differences seen between the risk groups. The results suggested that DEGs were primarily enriched in cell metabolic and immune function terms, including the GO terms organelle fission, nuclear division, and chromosome segregation (Figure 8B and Supplementary Table S7) and the KEGG pathway terms cell cycle, and cytokine–cytokine receptor interaction (Figure 8C and Supplementary Table S8).

Then, GSEA of the KEGG results showed apparent enrichment of the terms bladder cancer, ubiquitin-mediated proteolysis, cell cycle, DNA replication, and mTOR signaling pathway in the high-risk group (Figure 8D and Supplementary Table S9). In addition, GSEA based on HALLMARK pathways suggested that tumor hallmarks, such as E2F targets, MTORC1 signaling, MYC targets V1, NOTCH signaling, the P53 pathway, Pl3K/AKT/mTOR signaling, TGF BETA signaling, and WNT/BETA CATENIN signaling, were significantly enriched in the high-risk group (Figure 8E and Supplementary Table S10).

**FIGURE 9:**
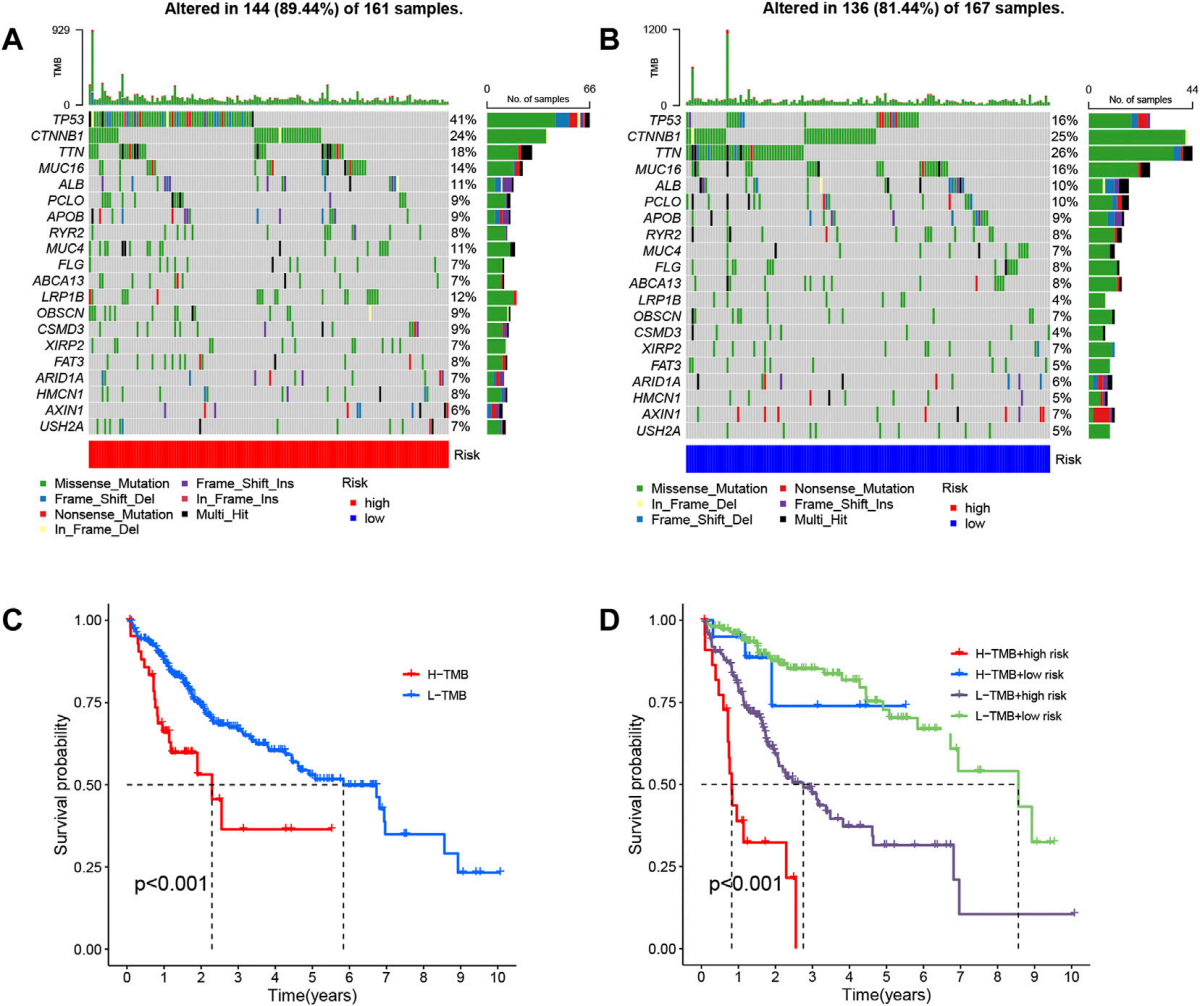
Analysis of gene alterations and their effects based on entire set. Differential gene mutations between the high- **(A)** and low-risk **(B)** groups were determined based on the long noncoding RNA (lncRNA) signature. **(C)** Patient overall survival (OS) is based on tumor mutational burden (TMB). **(D)** Patient OS over time based on TMB and risk score.

Moreover, we also evaluated differential gene mutations between the two risk groups in the entire set (Figures 9A, B). The results revealed that TP53 had the highest alteration rate, with a mutation rate of 41% in the high-risk group and 16% in the low-risk group.

The relationship between patient OS and tumor mutational burden (TMB) was analyzed, as depicted in Figure 9C, and the same analysis within risk groups is shown in Figure 9D. We found that samples with high TMB and high-risk had worse prognoses, and the risk score played the predominant role in deciding the outcome with increasing time.

#### 3.2.5 Identification of candidate drugs based on risk score

**FIGURE 10:**
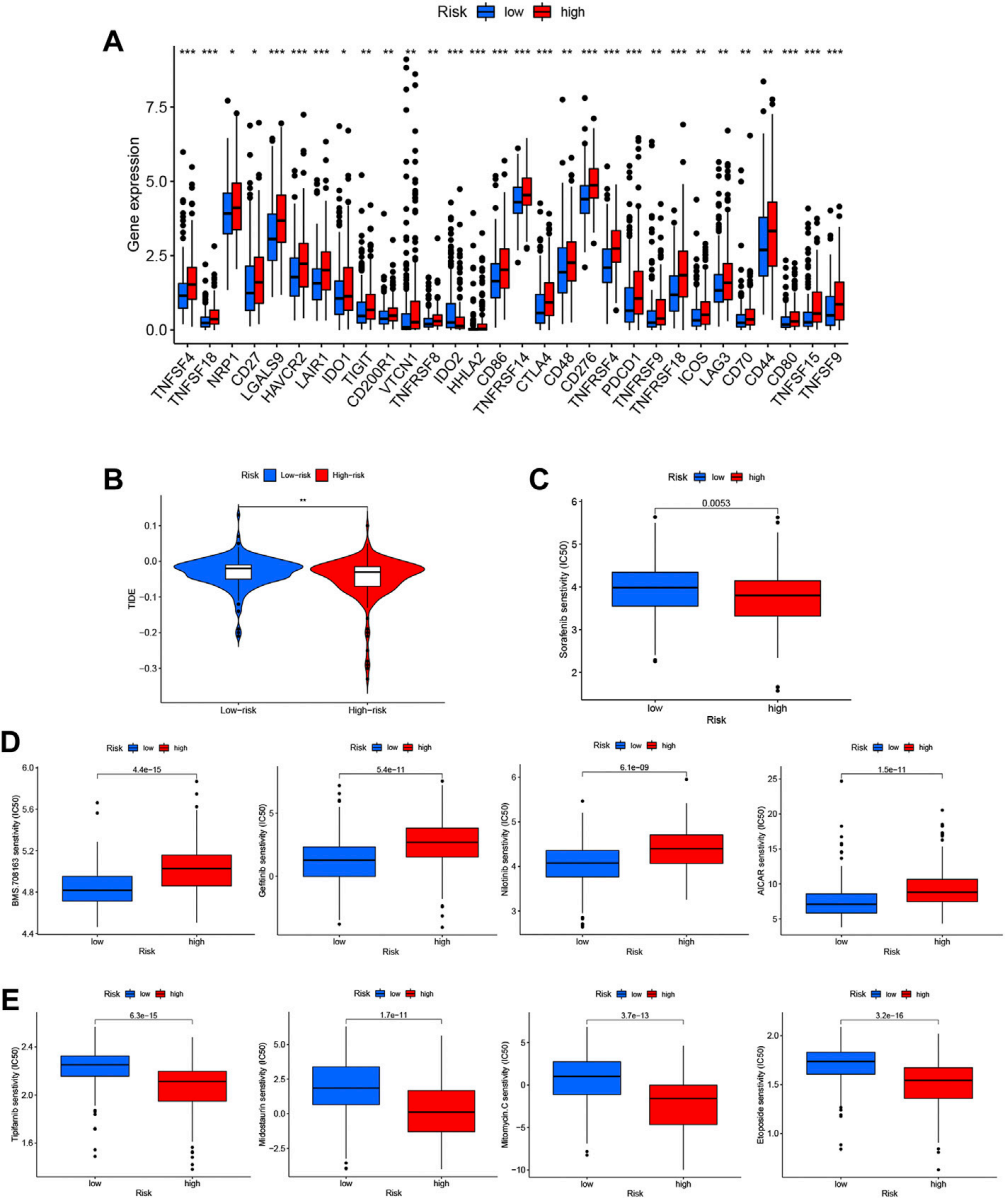
Identification of candidate drugs based on the N7-methylguanosine (m7G) risk score. **(A)** Immune checkpoint expression in the whole dataset. **(B)** Tumor immune dysfunction and exclusion (TIDE) analysis based on the risk score. **(C)** Analysis of sorafenib sensitivity based on the risk score. **(D)** Drugs with low IC50 values in the low-risk group. **(E)** Drugs with low IC50 values in the high-risk group.

Using the m7G-related lncRNA model, we evaluated the expression of immune checkpoints and activity levels in the whole case. There were considerable differences in immunological checkpoint expression between the two risk groups. The expression of *TNFSF4*, *TNFSF18*, *NRP1*, *CD27*, *LGALS9*, *HAVCR2*, *LAIR1*, *IDO1*, *TIGIT*, *CD200R1*, *VTCN1*, *TNFRSF8*, *HHLA2*, *CD86*, *TNFRSF14*, *CTLA4*, *CD48*, *CD276*, *TNFRSF4*, *PDCD1*, *TNFRSF9*, *TNFRSF18*, *ICOS*, *LAG3*, *CD70*, *CD44*, *CD80*, *TNFSF15*, and *TNFSF9* was higher, while that of IDO2 was considerably lower in the low-risk group (Figure 10A).

The tumor immune dysfunction and exclusion (TIDE) score (http://tide.dfci.harvard.edu) was calculated with each risk score group to analyze further the correlation between the risk score and immunotherapy sensitivity. The TIDE score can be used to predict a patient’s response to immunotherapy (Brinkman and van Steensel, 2019). The results showed that the TIDE score of the high-risk group was obviously lower than that of the low-risk group, suggesting that patients in the high-risk group may be more responsive to immunotherapy (Figure 10B).

We also analyzed the DEGs to identify potential treatments for HCC patients in the two risk groups. To accomplish this, 343 DEGs were entered into the CMAP database, and 56 drugs with distinct mechanisms of action were identified for further analysis. Patients in the low-risk group showed higher sensitivity to sorafenib, which is the current first-line systemic drug used in HCC patients (Figure 10C).

Additional medications predicted to be related to the m7G-related lncRNA signature and with a differential predicted appearance in response, which were manifested separately in the low-risk group (Figure 10D) and high-risk group (Figure 10E) should be investigated as potential therapeutics.

#### 3.2.6 Consensus clustering of N7-methylguanosine–related long noncoding RNAs and analysis based on subtype

**FIGURE 11:**
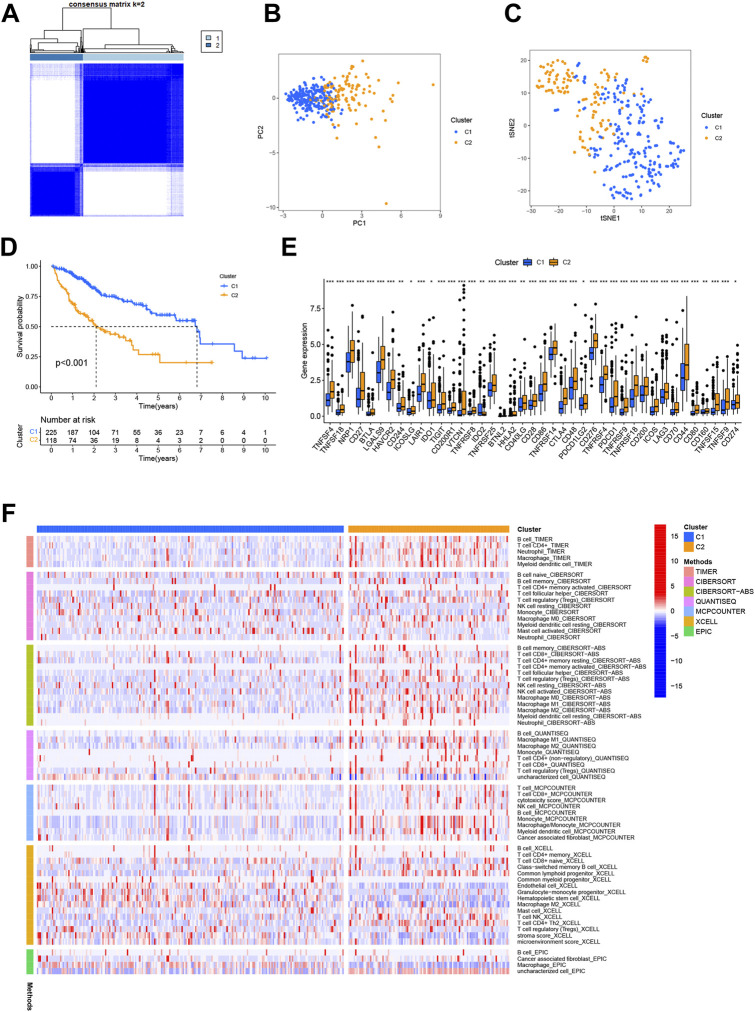
Consensus clustering of prognostic N7-methylguanosine (m7G)-related long noncoding RNAs (lncRNAs) and corresponding analysis. **(A)** Consensus matrix heatmap with 343 samples divided into Cluster 1 and Cluster 2. Principal component analysis (PCA) **(B)** and t-distributed stochastic neighbor embedding (t-SNE) analysis **(C)** were used to confirm the distinction between Cluster 1 and Cluster 2. **(D)** The Kaplan–Meier (K-M) curve analysis revealed the association between overall survival (OS) and risk score subtype. **(E)** Differential immune checkpoint gene expression in the two clusters. **(F)** Immune cell infiltration in the two clusters was assessed using the XCELL, TIMER, QUANTISEQ, MCPCOUNTER, EPIC, CIBERSORT−ABS, and CIBERSORT algorithms.

The molecular subtypes of HCC were explored using the NMF algorithm based on the expression of m7G-related lncRNAs. NMF clustering analysis was performed, and among k values of 2–9, 2 was shown to provide the best clustering reliability based on the cophenetic correlation coefficient. A total of 343 samples were separated into two clusters with clear boundaries in the matrix heatmap, which suggested robust clustering of the samples based on the lncRNA signature (Figure 11A). Information on the sample cluster is provided in Supplementary Table S11.

In addition, the PCA and t-distributed stochastic neighbor embedding (t-SNE) analysis confirmed that the two clusters could be distinguished (Figures 11B, C). The K-M curve analysis revealed a substantial survival difference between the groups when k = 2, with Cluster 1 having a better survival than Cluster 2 (*p* < 0.001) (Figure 11D).

We also detected the expression of multiple immune checkpoints in each subtype (Figure 11E) and immune cell infiltration in the two clusters generated with the above algorithm (Figure 11F). The expression of *TNFSF4*, *TNFSF18*, *NRP1*, *CD27*, *BTLA*, *LGALS9*, *HAVCR2*, *CD244*, *ICOSLG*, *LAIR1*, *IDO1*, *TIGIT*, *CD200R1*, *VTCN1*, *TNFRSF8*, *TNFRSF25*, *BTNL2*, *HHLA2*, *CD40LG*, *CD28*, *CD86*, *TNFRSF14*, *CTLA4*, *CD48*, *PDCD1LG2*, *CD276*, *TNFRSF4*, *PDCD1*, *TNFRSF9*, *TNFRSF18*, *CD200*, *ICOS*, *LAG3*, *CD70*, *CD44*, *CD80*, *CD160*, *TNFSF15*, *TNFSF9*, *CD274*, and *TNFSF9* was higher in Cluster 2, although IDO2 expression was significantly lower, somewhat consistent with the previous analyses. In addition, infiltration of various immune cells showed differences in the two clusters.

## 4 Discussion

HCC is the sixth most common malignancy and has a poor prognosis worldwide; HCC prognosis can be distinctly improved by early detection and treatment (Yarchoan et al., 2019). Identification of robust and meaningful biomarkers may improve prognosis. In addition, immunotherapy has dramatically improved patients’ quality of life with HCC, and many studies have shown objective results in patients receiving immune checkpoint inhibitors (Donisi et al., 2020).

Determining lncRNA-related modification mechanisms has become a common goal of current research (Wang et al., 2011; Rinn and Chang, 2012; Yang et al., 2014), and biological functions of lncRNAs (Esteller, 2011), such as binding with proteins, acting as protein sponges (Memczak et al., 2013), or serving as scaffolds that contribute to disease progression, have been revealed. Recent research has identified abnormal lncRNA expression as a diagnostic and prognostic biomarker in malignancies (Poursheikhani et al., 2020; Li et al., 2021; Wang H. et al., 2021).

In addition, in previous studies, many studies have investigated the ability of posttranscriptional regulation-associated genes to predict patient prognosis in various diseases, especially in malignancies, including breast cancer (Du et al., 2020), HCC (Li et al., 2020), and gastric cancer (Liu et al., 2021). LncRNA m7G modification is also important in gene regulation because it is necessary for expression (Juhling et al., 2009; Tomikawa, 2018). However, researchers have rarely explored the lncRNA m7G modifications as biomarkers of prognosis and immunotherapy response.

Here, data from 343 patients with OS time of more than 30 days were retrieved from TCGA to identify m7G-related prognostic lncRNAs.

As a result, an 11-m7G-related-lncRNA signature was generated, and the risk score calculated based on this signature was strongly associated with patient OS; the following lncRNAs were included in the risk signature: *AC026412.3*, *AC034229.4*, *AC091057.3*, *AC099066.2*, *AL049840.5*, *AL096678.1*, *AL158163.1*, *DUXAP8*, *GABPB1−AS1*, *LINC01224*, and *MKLN1−AS*. Multivariate Cox regression analysis was utilized to construct an ideal risk model. The high expression of *AC091057.3*, *AL158163.1*, and *MKLN1−AS* was found to be beneficial to patient OS, with negative correlation coefficients, and the remaining lncRNAs in the signature may promote cancer progression, having positive correlation coefficients. Some of the lncRNAs have already been confirmed to be significantly related to patient prognosis. Gu et al. (2021) found that suppression of *LINC01224* inhibited CRC cell proliferation, migration, and invasion while increasing apoptosis via the *LINC01224/miR-485-5p* axis. Qi et al. (2019) discovered that an elevated level of lncRNA *GABPB1-AS1* could inhibit *GABPB1* transcription, resulting in the dysregulation of the gene encoding the peroxiredoxin-5 (*PRDX5*) peroxidase and the suppression of cellular antioxidant capacity, implying that increased *GABPB1-AS1* levels may be linked to improved prognosis in HCC patients. In addition, Wang B. et al. (2021) found that *DUXAP8* promotes the Akt/mTOR signaling pathway, increasing tumor formation. However, the role of lncRNAs related to m7G modifications has been scarcely explored.

The identified lncRNAs were used to group patients into two risk groups by calculating a risk score, which was proven to be a competent, credible, and independent factor for assessing patient OS.

Given the importance of immunotherapy, the association between the risk score and immune cell infiltration was evaluated. Factors such as stromal cell abundance and enrichment of processes such as tumorigenesis and endothelial cell participation in the formation of new blood vessels and tumor microenvironment (TME) factor promotion of tumor aggressiveness have been shown to be relevant (Hida et al., 2004). The immune cell infiltration analysis showed intriguing results, with increased abundances of APCs, IDCs, macrophages, PDCs, and Th2 cells in the high-risk group and increased NK cells in the low-risk group, which may lead to the occurrence of an immunosuppressive TME. According to previous studies, Th2 cells cause immunosuppression that leads to tumor development (Mantovani et al., 2008). Another report indicated that the accumulation of M0 macrophages in the TME of HCC is a poor prognostic factor (Cai et al., 2022). In addition, APCs can benefit drug delivery to cancer cells and may minimize damage to healthy cells, serving as more applicable therapeutic agents with improved pharmacokinetic characteristics (Hafeez et al., 2020). In addition, the relationship between immunological scores and other immune functions was examined, and the low-risk group seemed to have a considerably higher IFN-II score. IFNs are essential components of the immune response to infections and cancers and influential promoters of the antitumor response (Dunn et al., 2006).

Co-inhibitory substances expressed by effector cells prevent the overactivation of immune checkpoints from delaying tumor development (Llovet et al., 2018; Cariani and Missale, 2019; Lee et al., 2020). Immune checkpoint inhibitors, including those targeting *PD-1*, *PD-L1*, *PD-L2*, and *CTLA4*, are considered the primary therapy for many malignant tumors (Donisi et al., 2020). In our study, there was a significantly higher level of *CTLA4* and *LAG3* expression in the high-risk group, and these markers are known to induce immunosuppression. Tregs have a constitutive expression of *CTLA-4*, which inhibits the immune response. *CTLA-4* competes with *CD28* for binding with *CD80/CD86*, thus decreasing activated T cells. At present, strategies to block immune checkpoints to prevent immune evasion, decrease Treg activity, and reactivate functions concerning antitumor immunotherapy are of interest (Inarrairaegui et al., 2018; Cariani and Missale, 2019). Further research of the other immune checkpoint-targeting agents with vast differences in predicted efficacy between the two groups may provide potential information for immunotherapy in HCC.

The GO and KEGG results indicate that the DEGs were primarily enriched in organelle fission, nuclear division, chromosomal segregation, cell cycle, and cytokine–cytokine receptor contact. Organelle fission, a type of cell transformation associated with organelle biology, plays an essential role in tumorigenesis because it is needed for adaptability to cellular and environmental changes and cancer therapies (Osteryoung, 2001). Repeat mistakes in chromosome segregation during mitosis cause chromosomal instability, a hallmark of cancer (Bakhoum et al., 2018). The cytokine–cytokine receptor interaction pathway preferentially induces inflammatory adaptive innate immunity, cell proliferation, cell differentiation, cellular damage, angiogenesis, and growth and restoration activities that attempt to restore equilibrium (Schreiber and Walter, 2010; Spangler et al., 2015).

Furthermore, the GSEA results depicted high enrichment of pathways and hallmarks related to tumor progressions in the high-risk group, such as cell cycle, bladder cancer, DNA replication, ubiquitin-mediated proteolysis, MTOR signaling pathway, TGF BETA signaling, E2F targets, MTORC1 signaling, P53 pathway, MYC targets V1, NOTCH signaling, Pl3K/AKT/mTOR signaling and WNT/BETA CATENIN signaling. These findings suggest that m7G lncRNA methylation plays an underlying regulatory role in HCC development. For example, proteolysis mediated by relevant cyclin chaperones and kinase inhibitors promotes the sequential activation of cyclin-dependent kinases. In recent years, studies have shown that dysregulation of the cell cycle induced by inefficient proteolytic control leads to uncontrolled cell proliferation and, consequently, the occurrence of tumors (Weinberg, 1995). E2F targets (E2Fs) are genes that encode a family of transcription factors related to the tumor progression in various cancers (Sozzani et al., 2006; Santos et al., 2014; Rennhack and Andrechek, 2015). In addition, mTOR signaling dysregulation has been linked to multiple human diseases, including obesity, diabetes, cancer, and neurological diseases (Cornu et al., 2013). At last, we analyzed the rates of gene alteration between the two risk groups, and TP53, one of the five most generally mutated genes in human malignancies today (Stratton, 2011), showed the highest mutation rate. Furthermore, the drug sensitivity analysis results showed that patients in the low-risk group were likely to have a different response to sorafenib, which has been the primary treatment for a decade, than those in the high-risk group.

We also separated the HCC patients into Cluster 1 and Cluster 2 using NMF clustering analysis. Most of the results were in line with those for the previously defined risk groups, suggesting the effectiveness and reliability of the risk score model for aiding individual treatment decisions in HCC.

In summary, our predictive model based on 11 m7G-related lncRNAs showed high clinical applicability and would cost substantially less than sequencing. The model also performed well in predicting the survival of HCC patients. Regardless, the study has several limitations. First, the model was constructed using TCGA data, and a patient cohort was not used. In addition, *in vivo* and *in vitro* tests are needed to confirm the findings. Moreover, the clinical guidelines for using this prognostic model need to be identified.

In conclusion, we used various methods to investigate the expression levels and prognostic value of m7G-related lncRNAs. We created an 11-lncRNA model with stand-alone prognostic utility in HCC. This is the first study to develop an m7G-related lncRNA risk model for HCC.

## 5 Conclusion

All the findings suggest that the developed m7G-related lncRNA signature can effectively assess patient OS and patient sensitivity to immunotherapy and other drugs, which may aid the discovery of novel immunotherapies and targeted therapies for HCC patients.

## Data Availability

The original contributions presented in the study are included in the article/Supplementary Material, and further inquiries can be directed to the corresponding author.

## Abbreviations

CI, concordance index; DEG, differentially expressed gene; GSEA, gene set enrichment analysis; GO, gene ontology; HCC, hepatocellular carcinoma; HR, hazard ratio; KEGG, kyoto encyclopedia of genes and genomes; K-M, Kaplan–Meier; LASSO, least absolute shrinkage and selection operator; OS, overall survival; PCA, principal component analysis; ROC, receiver operating characteristic; TCGA, the cancer genome atlas; t-SNE, t-distributed stochastic neighbor embedding.
